# Diagnostic accuracy of a point‐of‐care urine tenofovir assay, and associations with HIV viraemia and drug resistance among people receiving dolutegravir and efavirenz‐based antiretroviral therapy

**DOI:** 10.1002/jia2.26172

**Published:** 2023-09-21

**Authors:** Jienchi Dorward, Richard Lessells, Katya Govender, Pravi Moodley, Natasha Samsunder, Yukteshwar Sookrajh, Phil Turner, Christopher C. Butler, Gail Hayward, Monica Gandhi, Paul K. Drain, Nigel Garrett

**Affiliations:** ^1^ Nuffield Department of Primary Care Health Sciences University of Oxford Oxford UK; ^2^ Centre for the AIDS Programme of Research in South Africa (CAPRISA) University of KwaZulu–Natal Durban South Africa; ^3^ KwaZulu‐Natal Research and Innovation Sequencing Platform (KRISP) University of KwaZulu‐Natal Durban South Africa; ^4^ Africa Health Research Institute Durban South Africa; ^5^ Department of Virology University of KwaZulu‐Natal and National Health Laboratory Service, Inkosi Albert Luthuli Central Hospital KwaZulu‐Natal South Africa; ^6^ eThekwini Municipality Health Unit Durban South Africa; ^7^ Division of HIV Infectious Disease, and Global Medicine Department of Medicine University of California, San Francisco (UCSF) San Francisco California USA; ^8^ Department of Global Health, Schools of Medicine and Public Health University of Washington Seattle Washington USA; ^9^ Department of Medicine, School of Medicine University of Washington Seattle Washington USA; ^10^ Department of Epidemiology, School of Public Health University of Washington Seattle Washington USA; ^11^ Discipline of Public Health Medicine, School of Nursing and Public Health University of KwaZulu‐Natal Durban South Africa

**Keywords:** HIV, point‐of‐care, urine assay, tenofovir, adherence, viral failure

## Abstract

**Introduction:**

Novel point‐of‐care assays which measure urine tenofovir (TFV) concentrations may have a role in improving adherence monitoring for people living with HIV (PLHIV) receiving antiretroviral therapy (ART). However, further studies of their diagnostic accuracy, and whether results are associated with viraemia and drug resistance, are needed to guide their use, particularly in the context of the global dolutegravir rollout.

**Methods:**

We conducted a cross‐sectional evaluation among PLHIV receiving first‐line ART containing tenofovir disoproxil fumarate at enrolment into a randomized trial in two South African public sector clinics. We calculated the diagnostic accuracy of the Abbott point‐of‐care immunoassay to detect urine TFV compared to liquid chromatography‐tandem mass spectrometry (LC‐MS/MS). We evaluated the association between point‐of‐care urine TFV results and self‐reported adherence, viraemia ≥1000 copies/ml and HIV drug resistance, among people receiving either efavirenz or dolutegravir‐based ART.

**Results:**

Between August 2020 and March 2022, we enrolled 124 participants. The median age was 39 (IQR 34–45) years, 55% were women, 74 (59.7%) were receiving efavirenz and 50 (40.3%) dolutegravir. The sensitivity and specificity of the immunoassay to detect urine TFV ≥1500 ng/ml compared to LC‐MS/MS were 96.1% (95% CI 90.0−98.8) and 95.2% (75.3−100.0), respectively. Urine TFV results were associated with short (*p*<0.001) and medium‐term (*p* = 0.036) self‐reported adherence. Overall, 44/124 (35.5%) had viraemia, which was associated with undetectable TFV in those receiving efavirenz (OR 6.01, 1.27−39.0, *p* = 0.014) and dolutegravir (OR 25.7, 4.20−294.8, *p*<0.001). However, in those with viraemia while receiving efavirenz, 8/27 (29.6%) had undetectable urine TFV, compared to 11/17 (64.7%) of those receiving dolutegravir. Drug resistance was detected in 23/27 (85.2%) of those receiving efavirenz and only 1/16 (6.3%) of those receiving dolutegravir. There was no association between urine TFV results and drug resistance.

**Conclusions:**

Among PLHIV receiving ART, a rapid urine TFV immunoassay can be used to accurately monitor urine TFV levels compared to the gold standard of LC‐MS/MS. Undetectable point‐of‐care urine TFV results were associated with viraemia, particularly among people receiving dolutegravir.

**Trial registration:**

Pan‐African Clinical Trials Registry: PACTR202001785886049.

## 1 INTRODUCTION

Early identification and management of HIV viraemia among people living with HIV (PLHIV) receiving antiretroviral therapy (ART) is important to ensure rapid viral re‐suppression, which prevents the development of HIV drug resistance (HIVDR), morbidity and mortality, and HIV transmission [1]. Viraemia may be caused by drug−drug interactions, stockouts, side effects, inconsistent adherence to effective ART and/or HIVDR, but in low‐ and middle‐income countries (LMICs), resistance and drug‐level testing are not widely available [2], making management of viraemia more difficult for clinicians.

Tenofovir disoproxil fumarate (TDF) is a key component of World Health Organization‐recommended ART and is used by over 95% of people receiving ART in LMICs [3, 4], thereby serving as a potential target for objective adherence monitoring. TDF is metabolized to tenofovir (TFV), which is converted intracellularly to TFV‐diphosphate (TFV‐DP). TFV has a short plasma half‐life [5], is excreted renally and is readily measured in urine. TFV‐DP accumulates in red blood cells and has a longer half‐life [6] than plasma TFV. In studies among people without HIV using TDF for pre‐exposure prophylaxis [7], urine TFV concentrations have been associated with HIV seroconversion [8, 9]. However, measurement can require liquid chromatography‐tandem mass spectrometry (LC‐MS/MS), which is not feasible in many settings. Antibody‐based point‐of‐care urine TFV immunoassays have recently been developed for use in clinical settings to accurately detect TDF adherence within the past 2–5 days [10].

While several clinical trials are evaluating the impact of point‐of‐care urine TFV assays on HIV treatment outcomes [11, 12, 13], more observational data are needed to further evaluate diagnostic accuracy, and associations between point‐of‐care urine TFV results with HIV viraemia and drug resistance. In the context of the global rollout of the fixed‐dose combination of TDF‐lamivudine‐dolutegravir, objective measures of adherence may be even more useful. Dolutegravir is an integrase inhibitor with a high genetic barrier to resistance and emergent drug resistance has so far been rare [14], meaning viraemia is most likely secondary to non‐adherence. In contrast, previously recommended non‐nucleoside reverse transcriptase inhibitors (NNRTIs) (such as efavirenz) are more susceptible to the development of drug resistance, meaning viraemia may be caused by either poor adherence or drug resistance. Thereby, demonstrating good adherence in the presence of viraemia on efavirenz‐based ART may help identify people with drug resistance.

In this study, we aimed to evaluate the diagnostic accuracy of a point‐of‐care test to detect urine TFV at the manufacturer threshold of 1500 ng/ml, compared to the gold standard LC‐MS/MS. This threshold is estimated to classify 98% of people who took a TDF dose 24 hours ago as adherent, and 86% of those who last took a dose 96 hours ago as non‐adherent [15]. We also aimed to describe the association between point‐of‐care urine TFV results, and HIV viraemia, drug resistance and self‐reported adherence, among people receiving dolutegravir versus efavirenz. We hypothesized that a detectable point‐of‐care urine TFV result would be more strongly associated with viral suppression among people receiving dolutegravir compared to efavirenz, because of the lower likelihood of drug resistance with dolutegravir. We also hypothesized that in people with confirmed viraemia, a detectable urine TFV result would be associated with HIVDR among people receiving efavirenz.

## 2 METHODS

### 2.1 Study design

We conducted a prospective diagnostic accuracy sub‐study within the POwER study. POwER is an open‐label, individually randomized, feasibility study of point‐of‐care HIV viral load (VL) testing to enhance re‐suppression among people with HIV viraemia while receiving first‐line ART [16, 17].

### 2.2 Participants

We included all POwER participants who were taking TDF. PLHIV were eligible for POwER if they were receiving first‐line dolutegravir or efavirenz‐based ART and with recent viraemia >1000 copies/ml in the past 6 weeks, for which they had not yet received enhanced adherence counselling. Prior to the screening and enrolment visit, participants did not know that TFV drug levels would be measured. Some participants may have been asked to attend the clinic to review their recent blood results, but the majority did not know that their VL was high until the screening visit. At enrolment, participants provided socio‐demographic and clinical details, including self‐reported adherence, and had urine, dried blood spot (DBS) and plasma samples taken and stored at −80°C, for retrospective testing.

### 2.3 Test methods

#### 2.3.1 Point‐of‐care urine TFV testing

We tested thawed urine samples according to the manufacturer's instructions using the Abbott (Abbott) lateral flow point‐of‐care urine TFV assay. Specifically, 3–4 urine drops were added to the test well, with the result read by two independent laboratory technicians after 3–5 minutes. Photos of discrepant results were adjudicated by a third investigator.

#### 2.3.2 Reference standard urine TFV, and TFV‐DP concentrations

We used LC‐MS/MS at the Africa Health Research Institute in Durban to quantitate TFV levels in thawed urine samples, and measure TFV‐DP concentrations in DBS [18].

#### 2.3.3 VL and HIVDR

We tested VL with the cobas HIV‐1 assay (lower limit of quantitation 20 copies/ml) using the cobas 6800 platform (Roche) in the National Health Laboratory Service at the Inkosi Albert Luthuli Hospital in Durban. For all samples with VL ≥1000 copies/ml, we attempted sequencing of HIV‐1 pol (protease [PR], reverse transcriptase [RT] and integrase [IN]) at the KwaZulu‐Natal Research Innovation and Sequencing Plaform (KRISP). Following RNA extraction, we amplified PR, RT and IN genes using the amplification module of the Applied Biosystems HIV‐1 Genotyping Kit with Integrase (Thermo Fisher Scientific); and sequenced on the Illumina MiSeq platform (Illumina). We identified drug resistance mutations at >20% frequency using Stanford HIVdb (version 9.1).

The person conducting all the above tests was blinded to the results from other methods.

### 2.4 Statistical analysis

We assessed the diagnostic accuracy (sensitivity, specificity, and positive and negative predictive values) of the point‐of‐care TFV test to detect urine TFV at the manufacturer‐stated threshold of 1500 ng/ml. We compared self‐reported short‐term and longer‐term adherence with point‐of‐care urine TFV results using logistic regression models. We then described the proportions of PLHIV with and without viraemia ≥1000 copies/ml who had detectable and undetectable point‐of‐care urine TFV tests. To determine the usefulness of the urine TFV test to predict viraemia and suppression in this study population, we calculated the pre‐test probability (prevalence of viraemia and suppression before testing), and the post‐test probability (prevalence after stratification by urine TFV test result). Among participants with a VL ≥1000 copies/ml, we also assessed proportions with and without HIVDR who had detectable and undetectable point‐of‐care urine TFV results, and pre‐ and post‐test probabilities for drug resistance. We conducted the above analyses separately among participants receiving efavirenz or dolutegravir. We also conducted sensitivity analyses using a viraemic threshold of ≥50 copies/ml. Lastly, among people with unexpected urine TFV results based on viraemia and HIVDR results (e.g. viraemia, no HIVDR, but *detectable* point‐of‐care urine TFV), we assessed longer‐term adherence by describing TFV‐DP levels in DBS [6, 19].

The sample size was determined by the number of participants enrolled into POwER and receiving TDF. We analysed data using R 4.2.0 (R Foundation for Statistical Computing).

### 2.5 Ethical approvals

The eThekwini Municipality Health Unit Research Committee, the KwaZulu‐Natal Provincial Health Research Ethics Committee (KZ_202002_005), the University of KwaZulu‐Natal Biomedical Research Ethics Committee (BREC 00000836/2019) and the University of Oxford Tropical Research Ethics Committee (OxTREC 66‐19) approved the study. Written informed consent was obtained from all participants. POwER is registered on the Pan African Clinical Trials Registry (PACTR202001785886049).

## 3 RESULTS

### 3.1 Study population

**Figure 1:**
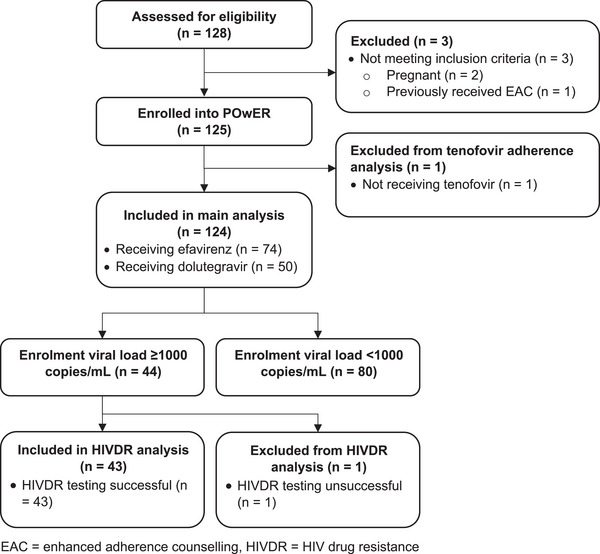
Flow diagram of POwER study participants. Abbreviations: EAC, enhanced adherence counselling; HIVDR, HIV drug resistance.

Between August 2020 and March 2022, we enrolled 125 participants into POwER (Figure 1); 124 were receiving TDF and were included in this analysis. The median age was 39 years (interquartile range [IQR] 34–45) and 68 (54.8%) were women (Table 1). Seventy‐four (59.7%) were receiving efavirenz for a median of 4.2 years (2.1–6.0), and 50 (40.3%) were receiving dolutegravir, for a median of 0.6 years (0.5–1.0). Median time since the pre‐enrolment viraemic VL was 15 days (13–21). In December 2020, we were informed that 45 participants had their pre‐enrolment VLs measured on a defective VL analyser which overestimated some VL results. Therefore, the viraemic sample used to determine eligibility may have been falsely high. After discussion with the ethics committees, we continued to include these participants in POwER and this sub‐study in order to have a wide range of VL results. At enrolment, 57/124 (46.0%) were suppressed <50 copies/ml, 23/124 (18.5%) had VL 50–999 copies/ml and 44/124 participants (35.5%) had viraemia ≥1000 copies/ml. Among the 43 with successful HIVDR testing, 24/43 (55.8%) had mutations conferring resistance to their current regimen. Among those receiving efavirenz, 23/27 (85.2%) had resistance to their current regimen, versus 1/16 (6.3%) of those receiving dolutegravir (M184V mutation alone). Overall, 23.4% self‐reported missing a dose in the past 4 days, and 62.9% reported last missing a dose over 4 weeks ago (Table 1).

**Table 1:** Baseline demographics of study population, *n* = 124

Variable	Levels	Total
Age, years	Median (IQR)	39.0 (34.0–45.0)
Gender	Women	68 (54.8)
	Men	56 (45.2)
Ethnicity	Black African	121 (97.6)
	Other	3 (2.4)
Time since ART initiation, years	Median (IQR)	4.1 (1.5–6.1)
Current ART regimen	TDF/FTC/EFV	74 (59.7)
	TDF/3TC/DTG	50 (40.3)
Time on current regimen, years	Median (IQR)	1.6 (0.7–5.0)
ART side effects	No	120 (96.8)
	Yes	4 (3.2)
Enrolment CD4 count category, cells/μl	<200	21 (16.9)
	200−349	25 (20.2)
	350−499	29 (23.4)
	≥500	49 (39.5)
Last time participant missed a dose of ART	<2 weeks	30 (24.2)
	2−4 weeks	16 (12.9)
	1−3 months	16 (12.9)
	>3 months	8 (6.5)
	Never	54 (43.5)
Number of ART doses missed in past 4 days	0	95 (76.6)
	1	14 (11.3)
	2	9 (7.3)
	3	2 (1.6)
	4	4 (3.2)
Point‐of‐care urine tenofovir test result	Not present	24 (19.4)
	Present	100 (80.6)
Urine tenofovir concentration, ng/ml	Median (IQR)	20,000.0 (7280.0–33,625.0)
Enrolment viral load, copies/ml	<50	57 (46.0)
	50–999	23 (18.5)
	≥1000	44 (35.5)
Any HIV drug resistance against current regimen?	No	19 (15.3)
	Yes	24 (19.4)
	Unsuccessful	1 (0.8)
	Viral load <1000 copies/ml	80 (64.5)

Abbreviations: 3TC, lamivudine; ART, antiretroviral therapy; DTG, dolutegravir; EFV, efavirenz; FTC, emtricitabine; IQR, interquartile range; TDF, tenofovir disoproxil fumarate.

### 3.2 Point‐of‐care urine TFV test

#### 3.2.1 Diagnostic accuracy

At enrolment, 100 (80.6%) had urine TFV detected with the point‐of‐care test. Median LC‐MS/MS urine TFV concentration was 20,000 ng/ml (IQR 7280–33,625). Compared to quantitative urine TFV concentrations, the point‐of‐care TFV test was accurate at detecting TFV at the manufacturer threshold of 1500 ng/ml, with a sensitivity of 96.1% (95% confidence interval [CI] 90.0−98.8) and specificity of 95.2% (75.3–100.0, Table 2). All five discrepant results occurred in samples with LC‐MS/MS TFV between 500 and 3000 ng/ml (Table S1).

**Table 2:** Analytic performance of the point‐of‐care tenofovir test to detect urine tenofovir at the manufacturer threshold of 1500 ng/ml

		LC‐MS/MS urine TFV (ng/ml)		
		*<1500*	≥*1500*	*Total*	
**POC TFV**	TFV not detected	20	4	24	
	TFV detected	1	99	100	
	*Total*	21	103	124	
Sensitivity		96.1% (90.0−98.8), *p*<0.001^*^			
Specificity		95.2% (75.3−100.0), *p*<0.001^*^			
PPV		99.0% (93.9−100.0), *p*<0.001^*^			
NPV		83.3% (63.4−93.8), *p* = 0.002^*^			

Abbreviations: LC‐MS/MS, liquid chromatography‐tandem mass spectrometry; NPV, negative predictive value; POC, point‐of‐care; PPV, positive predictive value; TFV, tenofovir.

* *p* Values testing the null hypothesis that the POC TFV test result has no relationship with urine TFV concentration (i.e. a sensitivity or specificity of 50%).

#### 3.2.2 Self‐reported adherence

Self‐reported missed doses in the past 4 days, and most recently self‐reported missed doses, were both associated with undetectable point‐of‐care urine TFV (Table S2). However, 12/95 (12.6%) who reported missing no doses in the past 4 days had undetectable urine TFV. Two were false negatives, with LC‐MS/MS concentrations >1500 ng/ml, and so were incorrectly classified as “non‐adherent” by the point‐of‐care test.

#### 3.2.3 Association with viraemia

Undetectable TFV on point‐of‐care urine testing was associated with viraemia ≥1000 copies/ml in people receiving efavirenz (odds ratio [OR] 6.01, 1.27−39.0, *p* = 0.014) and dolutegravir (OR 25.7, 4.20−294.8, *p*<0.001). Among those with viraemia ≥1000 copies/ml, 29.6% (13.8−50.2) of those receiving efavirenz had undetectable TFV, compared to 64.7% (41.1−82.7) of those receiving dolutegravir (Fisher's exact test for difference *p* = 0.031, Table 3). Among those with viral suppression <1000 copies/ml, 93.6% (82.0−98.4) of those receiving efavirenz and 93.9% (79.2−99.2) of those receiving dolutegravir had a detectable urine TFV.

**Table 3:** Association of point‐of‐care urine TFV results with viraemia, and HIV drug resistance

Viral load (copies/ml)							
		EFV only			DTG only		
		*<1000*	≥*1000*	*Total*	*<1000*	≥*1000*	*Total*
**POC TFV**	TFV not detected	3	8	11	2	11	13
	TFV detected	44	19	63	31	6	37
	*Total*	47	27	74	33	17	50
		%, (95% CI), *p*‐value^a^			%, (95% CI), *p*‐value^a^		
% with undetectable TFV, of those with viraemia		29.6 (13.8−50.2), 0.052			64.7 (41.1−82.7), 0.332^b^		
% with detectable TFV, of those suppressed		93.6 (82.0−98.4), <0.001			93.9 (79.2−99.2), 0.001^c^		
% with viraemia, of those with undetectable TFV		72.7 (42.8−90.5), 0.227			84.6 (56.3−96.6), 0.022^d^		
% suppressed, of those with detectable TFV		69.8 (57.5−79.8), 0.002			83.8 (68.4−92.6), 0.001^e^		

Presence of HIV drug resistance							
		*No HIV DR*	*HIV DR*	*Total*	*No HIV DR*	*HIV DR*	*Total*
**POC TFV**	TFV not detected	1	7	8	10	0	10
	TFV detected	3	16	19	5	1^f^	6
	*Total*	4	23	27	15	1	16
		%, (95% CI), *p*‐value^a^			%, (95% CI), *p*‐value^a^		
% with detectable TFV, of those with HIV DR		69.6 (48.9−84.4), 0.093			100 (17.0−100.0), 1.000		
% with undetectable TFV, of those without HIV DR		25.0 (4.0−71.0), 0.625			66.7 (41.5−84.8), 0.302		
% with HIV DR, of those with detectable TFV		84.2 (61.4−95.1), 0.004			16.7 (1.6−58.4), 0.219		
% without HIVDR, of those with undetectable TFV		12.5 (0.5−49.5), *p* = 0.070			100.0 (67.4−100.0), *p* = 0.002		

Abbreviations: ART, antiretroviral therapy; CI, confidence interval; DTG, dolutegravir; EFV, efavirenz; FTC, emtricitabine, #; HIVDR, HIV drug resistance; IQR, interquartile range; POC, point‐of‐care; TDF, tenofovir disoproxil fumarate; TFV, tenofovir.

^a^ *p* Values testing the null hypothesis that the POC TFV test result has no relationship with viraemia/HIVDR (e.g. of those with viraemia, 50.0% have positive POC TFV).

^b^ *p* for EFV versus DTG = 0.031.

^c^ *p* for EFV versus DTG = 1.00.

^d^ *p* for EFV versus DTG = 1.00.

^e^ *p* for EFV versus DTG = 0.639.

^f^ M184V mutation.

Among all people receiving efavirenz, the pre‐test probability was 36.5% (26.5−47.9) for viraemia and 63.5% (52.1−73.5) for viral suppression. After urine TFV testing, of those with undetectable urine TFV, the post‐test probability was 72.7% (42.8−90.5) for viraemia, and of those with detectable urine TFV, 69.8% (57.5−79.8) for viral suppression (Table 3). Among those receiving dolutegravir, the pre‐test probability was 34.0% (22.4−47.9) for viraemia and 66.0% (52.1−77.6) for viral suppression. Of those with undetectable urine TFV, the post‐test probability was 84.6% (56.3−96.6) for viraemia, and of those with detectable urine TFV, 83.8% (68.4−92.6) for viral suppression.

Using a ≥50 copies/ml threshold, undetectable point‐of‐care urine TFV remained associated with viraemia among those receiving efavirenz (*p* = 0.018) and those receiving dolutegravir (*p* = 0.002, Table S3), with 100% of those with undetectable TFV having viraemia.

#### 3.2.4 Association with HIVDR

Among 43 people with viraemia and successful HIVDR testing, detectable point‐of‐care urine TFV results were not associated with HIVDR against the current regimen in those receiving efavirenz (*p* = 1.000) or dolutegravir (*p* = 0.375). Of those receiving efavirenz, and with viraemia *≥*1000 copies/ml, 23/27 (85.2%, 66.7−94.6) had drug resistance to their current regimen, and of these 23, 16 had a detectable urine TFV (69.6%, 48.9−84.4, Table 2). Of those without HIVDR, 1/4 (25.0%, 4.0−71.0) had undetectable urine TFV. Among those receiving dolutegravir, only 1/16 (6.3%, 0.0−30.6) had drug resistance to their current regimen (M184V mutation). This one participant had a detectable urine TFV test, while 10/15 without HIVDR (66.7%, 41.5−84.8) had undetectable urine TFV. When looking at only drug resistance against nucleoside reverse transcriptase inhibitors (NRTIs), there was again no association with urine TFV results among those receiving efavirenz (*p* = 0.658) or dolutegravir (*p* = 0.375).

Regarding the ability of the urine TFV test to predict HIVDR among people with viraemia, among people receiving efavirenz, the pre‐test probability of HIVDR was 85.2% (66.7−94.6), and of not having HIVDR 14.8% (5.4−33.3). Nineteen people had detectable urine TFV despite viraemia, of whom 16 had HIVDR (post‐test probability of HIVDR 84.2%, 61.4−95.1). Eight had undetectable urine TFV, and of these, only one did not have HIVDR (post‐test probability of no HIVDR 12.5%, 0.5−49.5). Among people with viraemia and receiving dolutegravir, the pre‐test probability of HIVDR was 6.3% (0.0−30.6), and of not having HIVDR, 93.7%. Six had detectable urine TFV despite viraemia, of which only one had HIVDR (post‐test probability of HIVDR 16.7%, 1.6−58.4). Ten had undetectable TFV, and of these none had drug resistance (post‐test probability of no HIVDR 100%, 67.4−100.0).

#### 3.2.5 TFV‐DP levels

Among the eight participants with viraemia, no HIVDR and *detectable* urine TFV, one DTG participant had LC‐MS/MS urine TFV levels <1500 ng/ml, suggesting a false positive, detectable point‐of‐care urine TFV test. Of the remaining seven, urine TFV levels were >1500 ng/ml, suggesting recent TDF ingestion, but 6/7 had TFV‐DP levels <700 fmol/punch, which corresponds to poor longer‐term adherence with an estimated 0–3 tablets per week [6, 19] (Table S4A). Among the five participants with VL <1000 copies/ml, but *undetectable* urine TFV, two had LC‐MS/MSS urine TFV levels >1500 ng/ml, suggesting false negative, undetectable point‐of‐care urine TFV results. The remaining three participants had TFV‐DP levels between 200 and 550 fmol/punch, again suggesting inconsistent longer‐term adherence (Table S4B).

## 4 DISCUSSION

In this cross‐sectional study in South Africa, we demonstrate that a novel point‐of‐care test is accurate at detecting urine TFV, and identifies additional people with sub‐optimal adherence compared to self‐reported adherence measures. Furthermore, undetectable point‐of‐care urine TFV results were associated with viraemia, in particular among people receiving dolutegravir. Among people with viraemia, point‐of‐care urine TFV results were not associated with HIVDR.

We demonstrated an association between self‐reported adherence and urine TFV levels, and found that around 10% of people who reported missing no doses in the past 4 days had undetectable urine TFV using the point‐of‐care test. Therefore, this test could help identify people with unreported sub‐optimal adherence.

Undetectable urine TFV results were associated with viraemia, in particular among people receiving dolutegravir. Only 32% of those with viraemia while receiving efavirenz had an undetectable urine TFV; the majority had detectable urine TFV. However, this discrepancy can be explained by the high prevalence of HIVDR among people receiving efavirenz, meaning that drug resistance, rather than current poor adherence, was likely driving viraemia. Conversely, among people receiving dolutegravir, 63% of those with viraemia had undetectable urine TFV, suggesting that for these participants, poor adherence was the main cause of viraemia. This is supported by the low prevalence of HIVDR in participants on dolutegravir. Furthermore, among those with viraemia and a detectable urine TFV, TFV‐DP results suggested poor longer‐term adherence. In our study, the post‐test probability of viraemia or suppression at a threshold of 1000 copies/ml was over 80% among those receiving dolutegravir, suggesting that this test could be used to triage people receiving dolutegravir into different clinical pathways. At ≥50 copies/ml, undetectable urine TFV very strongly predicted viraemia, but detectable urine TFV performed less well at confirming viral suppression, likely because of the more sustained adherence required for suppression <50 copies/ml.

We did not find evidence of an association between urine TFV test results and HIVDR among people with viraemia (≥1000 copies/ml) receiving efavirenz or dolutegravir, which may be partly explained by the small sample size, meaning that estimates were not precise. However, the high prevalence of HIVDR among people receiving efavirenz (high pre‐test probability) meant that the test did not “add value,” as the post‐test probability of HIVDR remained similarly high. Likewise, the very low prevalence of resistance among people receiving dolutegravir (low pre‐test probability) meant that the test was again not helpful, with post‐test probabilities remaining similar.

There is one other published study assessing the analytic performance of the Abbot point‐of‐care TFV immunoassay compared to LC‐MS/MS, conducted as part of its development and validation. In 300 randomly selected stored urine samples from the TARGET TDF dosing trial [20], the point‐of‐care urine TFV assay had a sensitivity of 97% (95−99) and specificity of 99% (94−100) at a cut‐off of 1500 ng/ml [10]. Of note, all the discrepant results in our analysis were close to the 1500 ng/ml threshold.

Our findings are similar to recent studies which have assessed various point‐of‐care urine TFV assays, and associations with viraemia and/or HIVDR. A study from Lesotho among PLHIV receiving TDF‐based ART (95% on dolutegravir) found that the UrSure point‐of‐care test (now known as SureQuick Rapid Tenofovir Adherence Test [OraSure Technologies Inc.]) did not detect urine TFV in 1/8 (12.5%) with viraemia ≥1000 copies/ml, and detected urine TFV in 395/398 (99%) of those with viral suppression [21]. Of the eight with viraemia, seven were receiving efavirenz‐based ART, but HIVDR testing was not done.

A case−control study within the ADVANCE trial in South Africa matched 139 participants, recently initiated on ART and with rebound viraemia, with 53 non‐viraemic controls [22]. Sixty‐six percent of participants with viraemia ≥200 copies/ml had an undetectable SureQuick Rapid Tenofovir Adherence Test, and 100% of those with viral suppression had a detectable urine TFV, which is similar to our findings. Among the 42 with successful HIVDR testing, drug resistance was detected in 16.7% of those receiving dolutegravir, and 61.1% of those receiving efavirenz. Overall, a detectable urine TFV result was associated with NRTI resistance alone, but not NRTI/NNRTI resistance. This may reflect the persistence of NNRTI mutations in the absence of drug pressure, whereas NRTI mutations are more likely to be superseded by wild‐type virus if ART is not being taken.

Another South African study, among 113 people with previous viraemia or treatment interruptions, found that among people with viraemia ≥400 copies/ml, 64.7% of those receiving dolutegravir/boosted protease inhibitors had an undetectable Abbott point‐of‐care urine TFV result, compared to only 4.8% among those receiving efavirenz. Only one person receiving efavirenz had undetectable urine TFV [23]. Similar to our findings, among those receiving dolutegravir/boosted protease inhibitors, 85% of those with undetectable urine TFV had viraemia, and 85% of those with detectable urine TFV were suppressed. As in our study, the prevalence of HIVDR was high among people with efavirenz (18/20, 90%), and much lower among those receiving dolutegravir (5/16 = 31%), although associations between HIVDR and urine TFV results were not evaluated. Two other studies, focusing on people with viraemia while receiving efavirenz, found associations between positive point‐of‐care urine TFV results and HIVDR mutations, but similar to our findings, the prevalence of HIVDR mutations was high, and so the pre‐ and post‐test probabilities of HIVDR did not change much after a positive urine TFV test [18, 24].

This is one of the first evaluations of the analytic performance of the Abbot point‐of‐care urine TFV assay, beyond the validation conducted as part of the assay's development [10]. It is also the first to use both VL, HIVDR and longer‐term TFV‐DP results to evaluate the clinical utility of the assay. Our inclusion of people receiving dolutegravir is important given the ongoing dolutegravir rollout. Limitations of our study include the use of frozen urine samples for retrospective point‐of‐care testing by laboratory staff, the short period during which participants had been receiving dolutegravir and the relatively small sample size, particularly of people with viraemia and drug resistance, which resulted in wide confidence intervals and may affect the reliability of the findings. Further studies in different populations and with larger sample sizes, or meta‐analyses of similar studies, are required to generate robust estimates of associations between point‐of‐care urine TFV results, and viraemia and HIVDR. Our study design meant that we enrolled people a median of 15 days after first viraemia, meaning that adherence patterns could have changed, particularly if people had collected ART at the visit when blood was taken for the first viraemic VL. While participants were not told that their urine would be tested, they may have improved their adherence in anticipation of re‐attending the clinic, which may be reflected in the six participants with detectable urine TFV, but low TFV‐DP levels and viraemia. This cross‐sectional analysis also does not explore the relationship between current adherence measures and future outcomes.

Our findings provide further evidence supporting the use of point‐of‐care urine TFV immunoassays as objective indicators of adherence, and provide further insights into where they may be utilized in clinical practice. With widespread global TDF use, and recent data suggesting point‐of‐care urine TFV results are also associated with viraemia among people taking TFV alafenamide [25], the test could be useful in a wide range of settings. However, the moderate performance at predicting viraemia seen in our and other studies suggests that current point‐of‐care urine TFV assays cannot replace VL testing, but could be used as an additional test in‐between annual VL testing to monitor adherence and complement routine VL testing. People with undetectable urine TFV could then receive targeted adherence support and potential subsequent VL testing. This may be particularly useful in differentiated ART delivery programmes, where point‐of‐care urine TFV testing could aid adherence assessment at community ART pick‐up points. Urine TFV testing could also be used in the months immediately following ART initiation when VL is still suppressing, to identify people with early adherence issues and thereby facilitate targeted early adherence support. We are currently evaluating this approach in the STREAM‐HIV trial [11]. Lastly, among people with viraemia, reflex urine TFV testing could help distinguish poor adherence from HIVDR. While our findings suggest limited utility in identifying dolutegravir resistance while the prevalence remains low, further work is needed as the dolutegravir rollout matures to determine whether the point‐of‐care urine TFV test could be used as a triage test to determine whether people with viraemia should have HIVDR testing, versus enhanced adherence support.

## 5 CONCLUSIONS

Point‐of‐care urine TFV may be used to monitor ART adherence and predict viraemia, particularly in people receiving dolutegravir.

## COMPETING INTERESTS

Abbott provided the urine TFV assays at no cost. The authors have no other competing interests to declare.

## AUTHORS’ CONTRIBUTIONS

JD, PKD and NG conceived the study. RL, KG, PM and NS were responsible for laboratory testing. RL, YS, CCB and GH contributed to study design. MG developed the urine TFV assay. JD analysed the data and wrote the first draft of the manuscript. All authors critically reviewed and edited the manuscript and consented to final publication.

## FUNDING

This work is supported by grants from the Wellcome Trust PhD Programme for Primary Care Clinicians (216421/Z/19/Z), the University of Oxford's Research England QR Global Challenges Research Fund (0007365) and the Africa Oxford Initiative (AfiOx‐119). HIV drug resistance testing and drug concentration testing was funded by the National Institute for Health and Care Research (NIHR) Community Healthcare MedTech and In Vitro Diagnostics Co‐operative at Oxford Health NHS Foundation Trust (MIC‐2016‐018); GH, CCB and PT also receive funding from this award.

## DISCLAIMER

The views expressed are those of the author(s) and not necessarily those of the NHS, the NIHR or the Department of Health and Social Care. For the purpose of open access, the author has applied a CC BY public copyright licence to any Author Accepted Manuscript version arising from this submission. The University of Oxford is the study sponsor. The funders and sponsor had no role in study design, manuscript submission, or collection, management, analysis or interpretation of study data.

## Supporting information

Supporting Information

## Data Availability

Bona fide researchers will be able to request access to anonymized trial data by contacting the corresponding author.
