# Supplementary material for: Diagnostic accuracy of a point‐of‐care urine tenofovir assay, and associations with HIV viraemia and drug resistance among people receiving dolutegravir and efavirenz‐based antiretroviral therapy
Source: J Int AIDS Soc. 2023 Sep 21;26(9):e26172. doi: 10.1002/jia2.26172 (PMC10514373; doi:10.1002/jia2.26172)
Supplement: Supplementary file 1 — Supporting Information [file JIA2-26-e26172-s001.docx]

**SUPPLEMENTARY FILE**

**Contents**

[1. Table S1: Point-of-care urine TFV results for samples measured between 500- 3000ng/mL with LCMS-MS 2](#_Toc142034778)

[2. Table S2: Self-reported adherence compared to point-of-care urine TFV results 2](#_Toc142034779)

[3. Table S3: Analytic performance of the point-of-care urine tenofovir test to detect viraemia ≥50 copies/mL 3](#_Toc142034780)

[4. Table S4: Viral load, HIV drug resistance, urine TFV and TFV-DP results for participants with unexpected point-of-care tenofovir results 4](#_Toc142034781)

## Table S1: Point-of-care urine TFV results for samples measured between 500- 3000ng/mL with LCMS-MS

| **Participant** | **LCMS-MS Urine TFV concentration (ng/mL)** | **Point-of-care urine TFV result** | **Discrepant result?** |
| --- | --- | --- | --- |
| A | 613 | Present | Discrepant |
| B | 687 | Not Present | Not Discrepant |
| C | 697 | Not Present | Not Discrepant |
| D | 944 | Not Present | Not Discrepant |
| E | 1030 | Not Present | Not Discrepant |
| F | 1150 | Not Present | Not Discrepant |
| G | 1620 | Not Present | Discrepant |
| H | 2090 | Not Present | Discrepant |
| I | 2430 | Not Present | Discrepant |
| J | 2790 | Not Present | Discrepant |

LCMS-MS = Liquid chromatography tandem mass spectrometry

## Table S2: Self-reported adherence compared to point-of-care urine TFV results

| **Adherence variable** | | **POC TFV assay** | | |
| --- | --- | --- | --- | --- |
|  |  | **Not Present** | **Present** | **Odds ratio* (95% CI), P** |
| Number of ART doses missed in past 4 days | 0 | 12 (12.6) | 84 (88.4) | 0.44 (0.27-0.67)  P = <0.001 |
|  | 1 | 3 (21.4) | 11 (78.6) |  |
|  | 2 | 5 (55.6) | 4 (44.4) |  |
|  | 3 | 1 (50.0) | 1 (50.0) |  |
|  | 4 | 3 (75.0) | 1 (25.0) |  |
| Last time participant missed a dose of ART (weeks) | Never | 5 (9.3) | 50 (92.6) | 0.66 (0.49-0.87)  P = 0.036 |
|  | >12 | 1 (12.5) | 7 (87.5) |  |
|  | 4-12 | 4 (25.0) | 12 (75.0) |  |
|  | 2-4 | 3 (18.8) | 13 (81.2) |  |
|  | <2 | 11 (36.7) | 19 (63.3) |  |

*Binomial logistic regression models.

POC = point-of-care, TFV = tenofovir

## Table S3: Analytic performance of the point-of-care urine tenofovir test to detect viraemia ≥50 copies/mL

| **Viral load (copies/mL)** | | | | | | | | |
| --- | --- | --- | --- | --- | --- | --- | --- | --- |
|  | | | **EFV only** | | | **DTG only** | | |
|  | | *<50* | | *≥50* | *Total* | *<50* | *≥50* | *Total* |
| **POC TFV** | TFV not detected | 2 | | 9 | 11 | 0 | 13 | 13 |
|  | TFV detected | 38 | | 25 | 63 | 17 | 20 | 37 |
|  | *Total* | 40 | | 34 | 74 | 17 | 33 | 50 |
| % with undetectable TFV, of those with viraemia | | 26.5 (14.5-43.4), p=0.009 | | | | 39.4 (24.7-56.4), p=0.296* | | |
| % with detectable TFV, of those suppressed | | 95.0 (82.4-99.4), p<0.001 | | | | 100 (77.9-100), p<0.001^†^ | | |
| % with viraemia, of those with undetectable TFV | | 81.2 (51.0-95.7), p=0.065 | | | | 100 (72.9-100), p<0.001^‡^ | | |
| % suppressed, of those with detectable TFV | | 60.3 (48.0-71.4), p=0.130 | | | | 45.9 (31.1-61.6), p=0.743^§^ | | |

*p for EFV vs DTG = 0.469, ^†^p for EFV vs DTG = 1.00, ^‡^p for EFV vs DTG = 0.774, ^§^p for EFV vs DTG = 0.485

## Table S4: Viral load, HIV drug resistance, urine TFV and TFV-DP results for participants with unexpected point-of-care tenofovir results

| **ID** | **ART regimen** | **Viral load (cps/mL)** | **Drug resistance against current ART?** | **POC TFV result** | **Quantitative urine TFV (ng/mL)** | **Quantitative DBS TFV-DP (fmol/punch)** | **Comment** |
| --- | --- | --- | --- | --- | --- | --- | --- |
| 1. **Viraemia ≥1000 copies/mL** **with no drug resistance, but detectable POC urine TFV** | | | | | | | |
| 1 | TDF / FTC / EFV | 3550 | No | Detected | 26900 | 828 |  |
| 2 | TDF / FTC / EFV | 2570 | No | Detected | 62000 | 634 | Low TFV-DP |
| 3 | TDF / FTC / EFV | 1450 | No | Detected | 4080 | 219 | Low TFV-DP |
| 4 | TDF / 3TC / DTG | 4900 | No | Detected | 61200 | 90 | Low TFV-DP |
| 5 | TDF / 3TC / DTG | 2950 | No | Detected | 43100 | 477 | Low TFV-DP |
| 6 | TDF / 3TC / DTG | 11700 | No | Detected | 33800 | 573 | Low TFV-DP |
| 7 | TDF / 3TC / DTG | 14800 | No | Detected | 15000 | 0 | Low TFV-DP |
| 8 | TDF / 3TC / DTG | 3240 | No | Detected | 613 | 103 | False positive POC TFV |
| 1. **Viral suppression <1000 copies/mL but undetectable POC urine TFV** | | | | | | | |
| 9 | TDF / FTC / EFV | <50 | NA | Not Detected | 0 | 201 | Low TFV-DP |
| 10 | TDF / FTC / EFV | <50 | NA | Not Detected | 1620 | 821 | False negative POC TFV |
| 11 | TDF / FTC / EFV | 340 | NA | Not Detected | 2090 | 483 | False negative POC TFV |
| 12 | TDF / 3TC / DTG | 90 | NA | Not Detected | 236 | 522 | Low TFV-DP |
| 13 | TDF / 3TC / DTG | 112 | NA | Not Detected | 252 | 354 | Low TFV-DP |

ART = antiretroviral therapy, POC = point-of-care, TFV = tenofovir, DBS = dried blood spot, TFV-DP = tenofovir diphosphate
